# Peristaltic pumps adapted for laminar flow experiments enhance *in vitro* modeling of vascular cell behavior

**DOI:** 10.1016/j.jbc.2022.102404

**Published:** 2022-08-19

**Authors:** Javier Abello, Shreya Raghavan, Yvette Y. Yien, Amber N. Stratman

**Affiliations:** 1 Department of Cell Biology and Physiology, Washington University in St Louis School of Medicine, St Louis, Missouri, USA; 2 Department of Biomedical Engineering, Texas A&M University, College Station, Texas, USA; 3 Pittsburgh Heart, Lung and Blood Vascular Medicine Institute and Department of Medicine, University of Pittsburgh, Pittsburgh, Pennsylvania, USA

**Keywords:** laminar flow, pulsatile flow, endothelial cells, *in vitro* modeling, BSA, bovine serum albumin, EC, endothelial cell, HSC, hematopoietic stem cell, HUVEC, human umbilical vein endothelial cell, MC, mural cell, PBS-Tx, Triton X-100 in PBS, PFA, paraformaldehyde, VE, vascular endothelial

## Abstract

Endothelial cells (ECs) are the primary cellular constituent of blood vessels that are in direct contact with hemodynamic forces over their lifetime. Throughout the body, vessels experience different blood flow patterns and rates that alter vascular architecture and cellular behavior. Because of the complexities of studying blood flow in an intact organism, particularly during development, the field has increasingly relied on *in vitro* modeling of blood flow as a powerful technique for studying hemodynamic-dependent signaling mechanisms in ECs. While commercial flow systems that recirculate fluids exist, many commercially available pumps are peristaltic and best model pulsatile flow conditions. However, there are many important situations in which ECs experience laminar flow conditions *in vivo*, such as along long straight stretches of the vasculature. To understand EC function under these contexts, it is important to be able to reproducibly model laminar flow conditions *in vitro*. Here, we outline a method to reliably adapt commercially available peristaltic pumps to study laminar flow conditions. Our proof-of-concept study focuses on 2D models but could be further adapted to 3D environments to better model *in vivo* scenarios, such as organ development. Our studies make significant inroads into solving technical challenges associated with flow modeling and allow us to conduct functional studies toward understanding the mechanistic role of shear forces on vascular architecture, cellular behavior, and remodeling in diverse physiological contexts.

Perfusing 2D or 3D cultures of endothelial cells (ECs) with fluid is a popular technique to model the effects of blood flow shear stress forces on the vasculature (1, 2, 3, 4, 5, 6, 7, 8, 9, 10, 11, 12, 13, 14, 15, 16, 17, 18). *In vitro* systems have been built to mimic oscillatory, pulsatile, and laminar flow forces or patterns experienced by ECs along different regions of the vascular tree. Consistent with observations under physiological conditions, ECs cultured under laminar flow conditions align parallel to the direction of flow (5, 6, 14, 15, 16, 18, 19, 20, 21, 22, 23, 24, 25, 26, 27, 28, 29, 30, 31). Similarly, ECs experiencing pulsatile or oscillatory flow conditions are more haphazardly organized, or with a perpendicular alignment to the flow direction (5, 6, 14, 15, 18, 19, 20, 21, 22, 23, 24, 25, 26, 27, 28, 29, 30, 31).

Peristaltic pumps have often been utilized in system designs because of their simplicity, their relatively low cost, and their ability to keep aseptic conditions as the pumped liquid is fully contained and does not directly contact any mechanical parts of the system. However, the caveat of peristaltic pumps is their inherent pulsation, because of intermittent contact of the pump rollers with the tubing that carries the liquid. This creates a temporal change in flow velocity profiles differing from steady flow velocities in laminar flow (32), altering the forces experienced by the surrounding environment—in this case, the cells. In similar systems that require pumps to transport fluids, such as fuel injectors, the implementation of pulsation dampeners, devices that reduce the pulsation in fluids by compensating the discontinuity of the flow with an applied force of equal magnitude, is necessary to protect the integrity of the ducts that carry the liquid. There are many ways to generate dampeners, but the most common methods offset force by liquid displacement, pressure regulation valves, or diaphragms/elastomers (33, 34).

While significant research has been done to build reliable platforms capable of generating physiologic levels of laminar *versus* oscillatory or pulsatile shear forces, systems remain inaccessible to the broad vascular biology research community for a number of reasons. For many, the price of a full commercial system capable of altering flow patterns, rates, or type prevents use of this modeling technique. Microfluidic systems and raised reservoir low-angle inlet platforms, while fantastic at modeling steady-state laminar flow, remain complicated to fabricate or set up (35, 36). Peristaltic pumps are cost-effective alternatives but, as described, are best suited to mimic pulsatory/oscillatory flow conditions (33). To account for this, we have adapted and optimized a commercially available multihead peristaltic pump system that has its own controller software (37) to reproducibly perform steady-state laminar flow or pulsatile flow experiments (Fig. 1*A*). Here, we will outline the steps needed to create pulse dampeners from easy to access supplies that can attach to a peristaltic pump to generate laminar flow. Furthermore, we discuss our parameters for eliciting flow responses, standardization/validation of this pump system, and downstream analysis techniques for studying cell biology using this platform.

**Figure 1:**
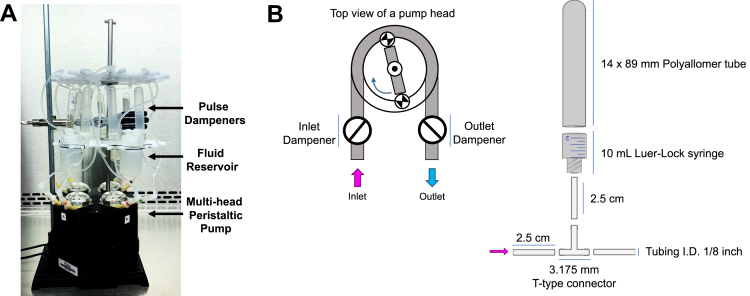
**Adaptations to a peristaltic pump to deliver laminar flow.***A*, picture of the flow system setup, including the four head peristaltic pump, dampeners, reservoirs, and flow chamber. *B*, *left panel*, shows the schematic representation of a peristaltic pump' head including the positions of the dampeners. The *right panel* is a schematic representation of how to assemble the dampeners.

## Results

### Generation of dampeners to offset pump pulsation

Addition of a dampener to a peristaltic pump is one way to offset the pulsatile forces generated by the mechanical properties of the pump. To do this, we utilized easy to access and cost-effective supplies to custom make small footprint dampeners to attach to any flow system (Fig. 1). To create the body of the dampener, the barrel of a 10 ml syringe (Becton Dickinson) was cut 12 mm from the base of the barrel. A thin wall polypropylene tube (Beckman) was bonded to the syringe barrel by applying a thin layer of two-part urethane adhesive (J-B Weld Company), following the manufacturer's recommendation. Because the internal diameter of the syringe barrel is 13.34 mm and the external diameter of the polypropylene tube is 14.43 mm, to assemble the dampener body, both parts must be forced together, which along with the adhesive avoids air or liquid leaks. The tubing connection to the dampener was created with 2.5 cm silicone tube (FisherBrand) inserted directly to the syringe Luer-Lok tip. At the other end of the silicone tubing, a T-type connector (Nalgene) was inserted (Fig. 1*B*).

Two dampeners are connected to the system, one on the inlet side and one on the outlet side of the system between the pump head and its respective media reservoir (Fig. 1*B*). The dampeners must be oriented vertically (with the dome of the polyallomer tube toward the ceiling) to ensure proper functioning, as they work by allowing liquid displacement into a closed chamber subjected to atmospheric pressure. If the dampeners are not properly oriented, laminar flow will not be achieved as bubbles will form within the system. Furthermore, if the chamber is inverted (with the dome oriented toward the floor), liquid will fill the dampener preventing its function. The media reservoirs (Fig. 1*A*) were constructed using 30 ml high-density polyethylene bottles (Nalgene). Two holes 4 mm in diameter were drilled in the caps of the high-density polyethylene bottles and two Elbow Luer Connector Male (ibidi) were bounded to the caps using the two-part urethane adhesive described previously. At the interior of the cap, two pieces of 2 and 4 cm silicone tubing were attached to the elbow connector as inlet and outlet ports, respectively.

Efficacy of the generation of laminar flow was confirmed two ways. First, we show *via* real-time visualization of quantum dots within the circulating media (Videos 1 and 3) that without the dampener (Video 1), there is a clear internal shift of the fluid/quantum dots as they transverse the flow chamber (flow is moving left to right across the video) from the peristaltic rollers stopping. This pulsation is virtually eliminated after addition of dampeners (Video 3). Second, we imaged the flow chambers using brightfield microscopy. Videos acquired at the level of the cells show a rhythmic pulsing of the fluid without the dampener (Video 2) that is abrogated by the addition of the dampener (Video 4).

**Figure 2:**
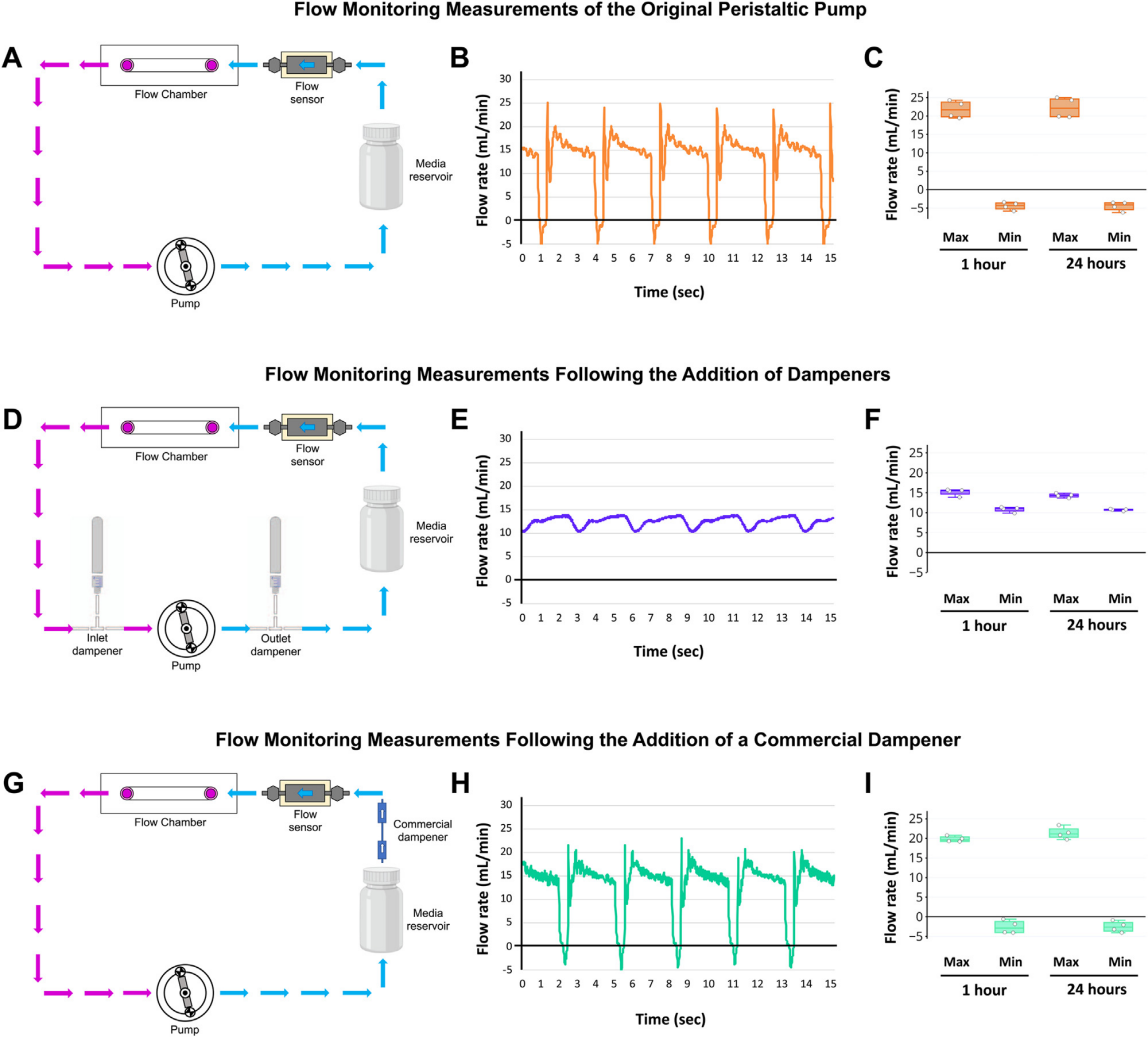
**Quantifying the effect of dampeners in offsetting pump pulsation.** A flow sensor was purchased to quantifiably measure the effects of dampeners in offsetting peristaltic pump pulsation. The flow sensor was placed directly prior to the flow chamber to ensure a representative measurement of flow forces felt by the endothelial cells (ECs) in the culture. *A*, schematic diagram of the flow circuit and placement of the flow sensor to measure flow forces generated by the original nonmodified peristaltic pump. *B*, pulse traces collected across 15 s of pump function, demonstrating marked pulsation of the fluid that is being flowed across the EC monolayer. *C*, average maximum and minimum flow forces generated by the endogenous function of the peristaltic pump at 1 and 24 h of culture. *D*, schematic diagram of the modified laminar flow circuit, including placement of our custom inlet/outlet dampeners and the flow sensor to measure forces generated by offsetting the pulsation coming out of the peristaltic pump. *E*, pulse traces collected across 15 s of pump function, demonstrating laminar flow (*i.e.*, sixfold suppression of fluid pulsation) across the EC monolayer. *F*, average maximum and minimum flow forces generated by the modified laminar function of the peristaltic pump at 1 and 24 h of culture. *G*, schematic diagram of the modified laminar flow circuit, including placement of a commercial dampener and the flow sensor to measure forces generated by offsetting the pulsation coming out of the peristaltic pump. *H*, pulse traces collected across 15 s of pump function, demonstrating mild suppression of fluid pulsation across the EC monolayer. However, as shown, our dampeners suppressed pulsation to a much higher degree (commercial 1.4-fold from peristaltic; lab-built sixfold from peristaltic). *I*, average maximum and minimum flow forces generated after commercial dampener modifications of the peristaltic pump at 1 and 24 h of culture. The box plots are graphed showing the median *versus* the first and third quartiles of the data (the *middle*, *top*, and *bottom lines* of the *box*, respectively). The *whiskers* demonstrate the spread of data within 1.5 × above and below the interquartile range. All data points are shown as discrete dots (averages from each of the four individual pumps).

To quantify the effectiveness of the dampeners in offsetting the inherent pulsatile nature of the pump, we installed a liquid flow sensor to the silicone tubing (SENSIRION) enabling us to determine directionality of flow (positive flow moving left to right across the sensor, negative if flow is moving right to left across the sensor) and the dynamic liquid flow rates of the system (Fig. 2). The sensor was installed before the inlet attachment point of the μ-Slide I 0.4 Luer slide of the flow circuit (Fig. 2*A*). The system was evaluated under the following conditions: (1) the flow system without dampeners, using the original function of the pump (pulsatile, Fig. 2, *A*–*C*); (2) the flow system modified with dampeners installed at the inlet and outlet of the pump head (laminar, Fig. 2, *D*–*F*); and (3) the flow system modified with a commercial dampener (SENSIRION) installed before the sensor as is recommended by the manufacturer (laminar, Fig. 2, *G*–*I*). The system was placed under our standard experimental conditions (5% CO_2_, 37 ^°^C, and 95% humidity for 24 h) and 15 s of sensor measurements of fluid flow collected at 1 and 24 h of culture. The 1- and 24-h time points were chosen to demonstrate the stability of flow forces across the time course of experimentation. The nonmodified pump as purchased has an inherent rhythmic pulsation to it (Fig. 2*B*), which can be dramatically offset by the incorporation of dampeners into the flow circuit (Fig. 2*E*). As a direct comparison, we tested the performance of a commercial SENSIRION dampener and demonstrate that our homemade dampeners were able to outperform a commercial version and suppress pulsation more dramatically (Fig. 2, *E* and *H*). While there is still minimal pulsation within the system, it is reduced approximately sixfold.

To account for variance in location of the dampeners between the commercial and homebuilt setups, we analyzed three different variations of the original commercial setup (Fig. 2*G*). Our first modification added a flat bottom fluidic restrictor with an internal diameter of 0.5 mm (provided with the flow sensor)—we call this variation V.1 (Fig. S1, *A*-*C*). Second, we installed the flow sensor with the restrictor between the pump outlet and the media reservoir. The commercial SENSIRION dampener remained located between the media reservoir and the inlet of the ibidi chamber (μ-Slide I 0.4 Luer)—we call this variation V.2 (Fig. S1, *D*-*F*). Third, we moved the commercial SENSIRION dampener to be between the pump outlet and the media reservoir, as we have it installed with our homebuilt dampener, leaving the flow sensor with the restrictor between the outlet of the media reservoir and the inlet of the ibidi chamber slide—we call this variation V.3 (Fig. S1, *G*-*I*). As shown in Fig. S1, none of these modifications significantly improved the performance of the commercial SENSIRION dampener by more than approximately 1%.

The small improvement obtained with variation V.2 by the addition of the restrictor to the flow sensor (Fig. S1, *D*-*F*) prompted us to test this modification in line with our homemade dampeners and reassess flow pulsation (Fig. 2*D*). Following the addition of the flat bottom fluidic restrictor to the flow sensor on our homemade dampener system, we observed an additional 15% decrease in flow pulsation as compared with our system as originally conceived without the restrictor (Fig. S2). All data presented throughout the remainder of the article utilize our custom dampeners and system as shown in Fig. 2, as this platform reliably allowed us to observe phenotypic differences in EC behavior in response to various types of flow stimuli without the purchase of additional commercial products. However, if higher levels of pulse dampening are desired, addition of a SENSIRION restrictor to the system can improve overall function of our homebuilt dampeners.

### Validation of cellular behavior in flow assays *via* live imaging analysis

To confirm that altered cellular behaviors are generated in response to differing flow stimuli from our modified pump system, we set up 2D live imaging assays to longitudinally track cellular motility and alignment over a 24-h period. Human umbilical vein ECs (HUVECs) or human aortic ECs were grown to confluence (2.5 × 10^5^ cell/slide) on 1 mg/ml gelatin-coated μ-Slide I 0.4 Luer slides in 1 × M199 media supplemented with 20% fetal bovine serum, 25 μg/ml of EC growth supplement, 0.01% heparin sodium salt, and 1 × antibiotic–antimycotic at 5% CO_2_, 37 ^°^C, and 95% humidity. The slides were then acclimated to a microscopy system containing a climate-controlled stage top incubator (EVOS M7000) to be cultured under conditions of constant laminar flow, at 12 to 15 dyn/cm^2^/s, or constant pulsatile flow, at 12 to 15 dyn/cm^2^/s and 60 RPM, for 24 h. To help maintain cell health, at the start of the experiment, the rate of flow was stepwise increased 10% every 10 min until reaching the desired final experimental flow rate.

**Figure 3:**
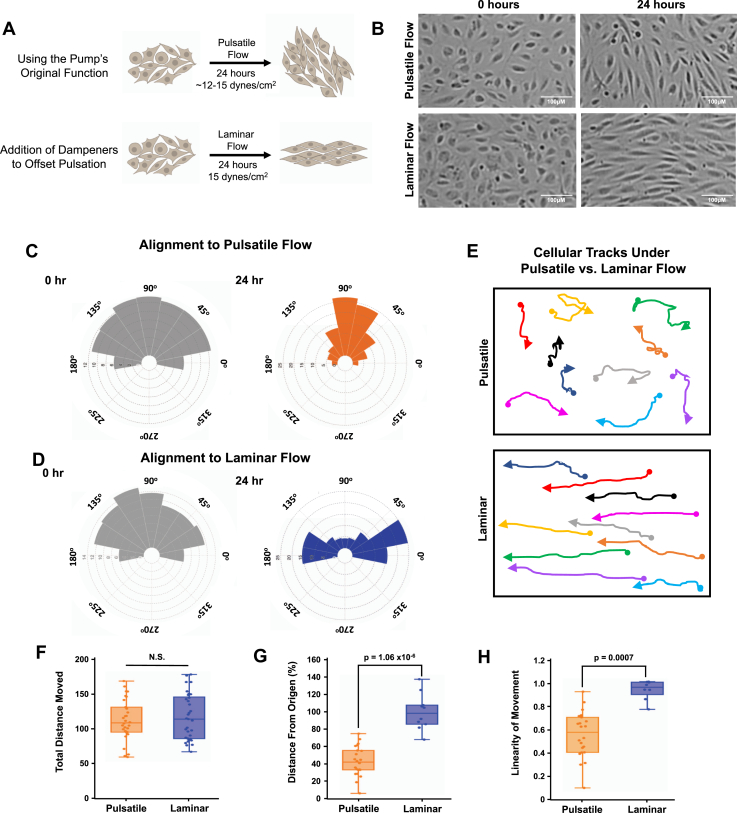
**Phenotypic characterization of pulsatile *versus* laminar flow conditions.***A*, a *cartoon* of the cell alignment expected under pulsatile and laminar flow conditions (*i.e.*, with and without dampeners). *B*, microscopy images show HUVEC alignment under pulsatile *versus* laminar flow conditions at 0 and 24 h as imaged utilizing our stage top incubated microscope system. *C* and *D*, distribution angles of HUVECs, showing the orientation of the cell’s longest axis relative to the direction of flow at 0 h (*gray*) and after 24 h (*orange/blue*) of pulsatile (*C*) *versus* laminar (*D*) flow. The alignment of the cells was carried out using the ImageJ plugin Directionality analysis, version 2.3.0, created by Jean-Yves Tinevez (https://imagej.net/plugins/directionality) implementing the method of Fourier components. n = 8 images, all cells within the image were included in the analysis. Data plotted using a bin size of 22.5^°^. The radial axis represents the proportion of cells that fall into each bin. *E*, representative cell tracks of HUVECs across the 24-h period of pulsatile (*top*) or laminar (*bottom*) flow. *Arrowheads* represent the direction of cellular movement, and *circles* represent the starting point of individual cells. *F*–*H*, average total distance traveled (*F*), average distance from origin (*G*, calculated based off of the linear distance between the starting point and ending point of individual cells), and linearity of movement (*H*, calculated as the ratio of *G* to *F*) shown for pulsatile *versus* laminar flow over the 24 h period of treatment. For *H*, linearity of movement: “1” is a movement path that is completely linear, whereas “0” is a movement path that is completely nonlinear, that is, curved. The box plots are graphed showing the median *versus* the first and third quartiles of the data (the *middle*, *top*, and *bottom lines* of the *box*, respectively). The *whiskers* demonstrate the spread of data within 1.5 × above and below the interquartile range. All data points (individual cells; n > 8) are shown as *discrete dots*, with outliers shown above or below the *whiskers*. Data are representative of three independent replicates. HUVEC, human umbilical vein endothelial cell.

Utilizing a microscope with a stage top incubator allows us to carry out multipoint time-lapse image acquisition. Images were acquired every 20 min for 24 h to follow dynamic changes in cell shape and alignment over time (Videos 5 and 6, Fig. 3, *A* and *B*). At the end of the experiment, individual images were assembled into a video file to watch cellular behavior across the full 24 h of imaging. Angle of alignment (Fig. 3, *C* and *D*), cellular tracking (Fig. 3*E*), and total distance moved/linearity of movement (Fig. 3, *F*–*H*) amongst other features can be analyzed from these videos utilizing free ImageJ/Fiji software (38). Our results show that in response to laminar flow, ECs align to and move against the direction of flow over the 24 h imaging period (Fig. 3, *D* and *E*, Video 6), whereas ECs subjected to pulsatile flow largely remain perpendicular to the direction of flow and move in a more haphazard fashion over the 24-h imaging period (Fig. 3, *C* and *E*, Video 5). Under both flow conditions, HUVECs on average move the same total distance; however, under laminar flow, HUVECs exhibit a larger linear displacement from their starting point of origin and move more linearly (Fig. 3, *F*–*H*). This behavior mimics what is seen with both *in vivo* and *in vitro* flow models (5, 6, 14, 15, 16, 19, 20, 21, 22, 23, 24, 25, 26, 27, 28, 29, 30, 31), validating that our system does indeed reliably model flow stimuli to elicit the expected changes in cellular behavior.

From a practical standpoint, use of this system does not require live imaging. If desired, the entire setup can be easily placed in a standard 37 ^°^C, 5% CO_2_, humidified tissue culture incubator. The peristaltic pump we adapted (Flocel (37)) has four pressure heads. When carrying out live imaging, stage top space prevents use of all four heads in tandem. Therefore, we often use the system in a standard incubator to allow conversion of two pressure heads to carry laminar flow, whereas two remain pulsatile to optimize throughput and pair culture conditions within a single experiment. At the end of the experiment, the cells can be collected for biochemical/molecular analysis to determine the downstream molecular impacts of individual flow stimuli: that is, rinsed with 1 × PBS and fixed with 4% paraformaldehyde (PFA) for immunostaining applications (discussed later), flushed with TRIZOL for mRNA collection, or lysed in sample buffer for protein collection (30).

### Immunostaining of cultures following flow

Following termination of flow, our method enables analysis of protein localization, cellular signaling, or mRNA composition, among other parameters, to interrogate downstream signaling pathways that occur as a consequence of flow. Here, we will outline our immunostaining protocol to analyze protein accumulation and localization in response to flow forces.

After 24 h of exposure to flow stimuli, the cultured ECs were rapidly rinsed twice with 3 ml 1 × PBS by flushing the slides using a 3 ml syringe. The cells were fixed in 100 μl of 4% PFA for at least 4 h before starting the immunostaining protocol. Following fixation, the PFA solution is removed from the slides and the cells rinsed three times with ice-cold 1 × PBS. If desired, the cultures can then be incubated with 100 μl of 0.1% Triton X-100 in 1 × PBS (PBS-Tx) for 10 min at room temperature to permeabilize the cells and stain for intracellular proteins. Next, the cells were incubated in 100 μl blocking buffer (1% bovine serum albumin [BSA] and 0.3 M glycine in 1 × PBS-Tx) at room temperature for 1 h. A primary antibody is chosen for the desired protein of interest and is diluted into 1% BSA/1 × PBS-Tx for incubation overnight at 4 °C. The next morning, the primary antibody is removed, and the cells rinsed with 1 × PBS-Tx three times to decrease nonspecific antibody binding. Secondary antibody is then added at a 1:2000 dilution in 1% BSA/1 × PBS-Tx, and the slides were incubated for 1 h at room temperature. Finally, Hoechst dye is added at a 1:5000 dilution and incubated for 30 min at room temperature for staining of DNA/nuclei. Three washes with PBS-Tx were done at the end of all the steps to remove excess antibody and decrease nonspecific background staining prior to mounting the slides for imaging analysis. Images were acquired utilizing a 20, 40, and 60 × objectives on a Nikon Ti2 inverted microscope equipped with a CSU-W1 confocal spinning disk (Yokogawa).

**Figure 4:**
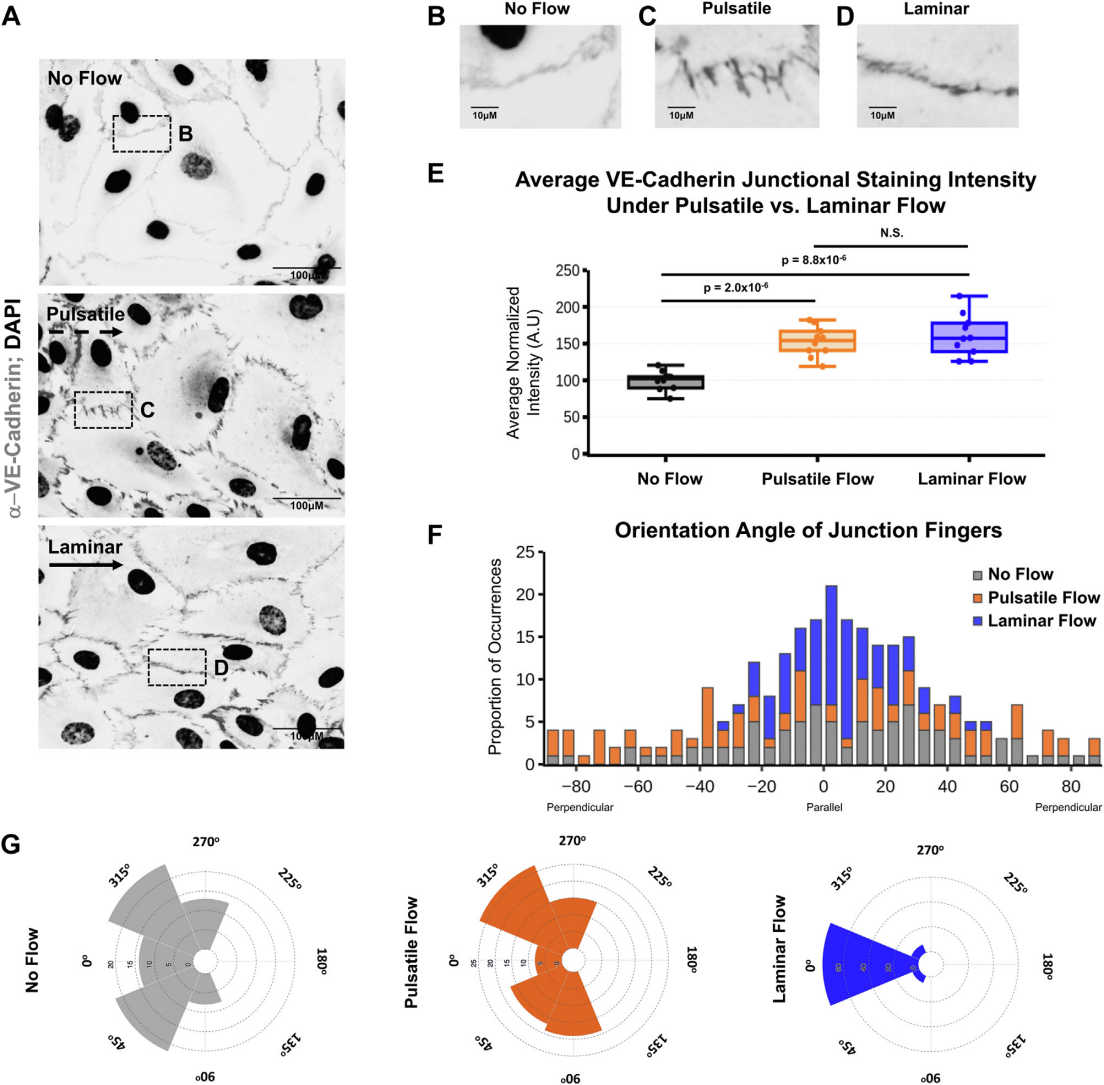
**Immunostaining the EC junctional marker, VE-cadherin, under varying flow conditions.***A*, representative microscopy images of cells immunostained for VE-cadherin (*grey*) and nuclei (*black*) under no flow, pulsatile flow, or laminar flow conditions. *B*–*D*, zoomed in imaged of the *white boxes* shown in *A*, showing changes in localization and intensity of VE-cadherin staining depending on flow forces applied to the EC monolayer. *E*, quantification of VE-cadherin staining intensity at the EC junction. *Black bar* shows the no flow condition, *orange bar* shows staining intensity following exposure to pulsatile flow (original pump function), and *blue bar* shows staining intensity following exposure to laminar flow (condition with dampeners). *F* and *G*, histogram (*F*) and radial histograms (*G*) depicting orientation angles of the VE-cadherin positive junctional “fingers” relative to the direction of flow (flow applied from *left* to *right* in images). No flow (*gray*), pulsatile flow (*orange*), and laminar flow (*blue*). The distribution of data shows that under no flow conditions, VE-cadherin fingers, if present, are randomly distributed. Under pulsatile flow conditions, these fingers skew to have a slightly more perpendicular orientation, when considering junctions parallel to the direction of flow (*i.e.*, “top” and “bottom” of the cells). Under laminar flow conditions, the fingers have a parallel orientation, when considering the junctions parallel to the direction of flow (*i.e.*, “top” and “bottom” of the cells). See Fig. S3 for schematic description. The alignment of the VE-cadherin fingers was carried out using the module for quantitative orientation measurement of the ImageJ plugin OrientationJ created by Daniel Sage (http://bigwww.epfl.ch/demo/orientation/). n = 45 cells, five images. Data plotted using a bin size of 45^°^ and are representative of two independent experiments. *F* and *G*, represent varying presentation of the same dataset to demonstrate different visualization techniques, (*F*) the *y*-axis represents the proportion of cells that fall into each bin; (*G*) the radial axis represents the proportion of cells that fall into each bin. The box plots are graphed showing the median versus the first and third quartiles of the data (the *middle*, *top*, and *bottom lines* of the *box*, respectively). The *whiskers* demonstrate the spread of data within 1.5 × above and below the interquartile range. All data points (average intensity from individual images; n > 8) are shown as individual dots. *p* values are indicated above statistically significant datasets and were generated using one-way ANOVA. Data are representative of three independent replicates. EC, endothelial cell; VE, vascular endothelial.

**Figure 5:**
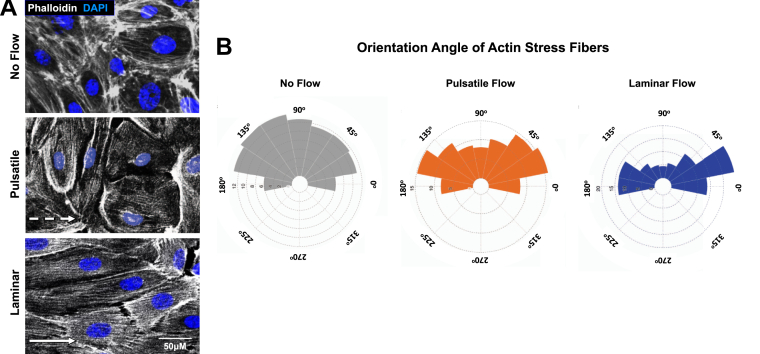
**Immunostaining of F-actin filaments with phalloidin-555 under pulsatile and laminar flow**. *A*, images of F-actin filaments (*white*) and nuclei (*blue*) under no flow (*top panel*), pulsatile flow (*middle panel*), and laminar flow (*lower panel*). *B*, orientation angle of the actin filaments relative to the direction of the flow (flow applied from *left* to *right*). No flow, (*gray*), pulsatile flow (*orange*), and laminar flow (*blue*). Under pulsatile flow conditions, the actin filament angle of orientation is randomly distributed, though skewed in orientation toward the direction that flow is coming from, while under laminar flow, for the actin filaments align more narrowly to be parallel to the direction of flow. The alignment of the F-actin filaments was carried out using the ImageJ plugin Directionality analysis, version 2.3.0, created by Jean-Yves Tinevez (https://imagej.net/plugins/directionality) implementing the method of Fourier components. n = 8 individual images, all cells within the image were included in the analysis. Data plotted using a bin size of 22.5^°^. The radial axis represents the proportion of cells that fall into each bin. Data are representative of three independent replicates.

As an example (Fig. 4), the EC junctional protein vascular endothelial (VE)-cadherin was labeled (1:100 dilution of primary antibody) and visualized using an Alexa Fluro-488 secondary antibody (1:2000 dilution, *green*). Nuclei (Hoechst, 1:5000 dilution) labeling is in *blue*. As shown, application of flow forces across ECs enhances the accumulation of VE-cadherin protein at junctions compared with no flow controls (Fig. 4, *A*–*D*). While no differences were noted in immunostaining intensity between laminar and pulsatile flow conditions (Fig. 4*E*), marked differences in orientation of VE-cadherin “fingers” were noted at junctional planes parallel to the flow direction (Fig. 4, *F* and *G*) (39). When quantified (orientation at 0^°^ being exactly parallel to flow and +90^°^ or −90^°^/270^o^ being perpendicular to flow), these fingers orient randomly under the no flow condition, with a slight increase in orientation at 45^°^ and 315^°^. The proportion of fingers increases in the 45 to 90^°^ and 270 to 315^°^ orientation in response to pulsatile flow conditions, whereas the fingers largely align parallel to the direction of flow (0^°^, ±25^°^) under laminar flow conditions. Furthermore, phalloidin staining was done to assess orientation of actin stress fibers in response to various flow forces (Fig. 5). As shown in both the representative images and the quantification, under the no flow and pulsatile flow conditions, the actin stress fibers are oriented randomly, with a slight skew in stress fiber orientation toward the direction of flow under the pulsatile flow condition. Conversely, under laminar flow conditions, the actin fibers are oriented parallel to the direction of flow.

## Discussion

In this work, we outline the validation and use of a peristaltic pump system that can be easily modified to concurrently deliver laminar or pulsatile flow to adjacent EC cultures in a highly reproducible manner. The system provides a cost-effective easy to use alternative for laboratories looking to conduct *in vitro* flow modeling experiments. Moreover, the platform allows for real-time imaging and analysis of cellular responses to flow (Fig. 3), coupled with downstream molecular analysis (30) (Figure 4, Figure 5).

Pulse dampeners are widely used to mitigate the interrupted pulsatile flow patterns, which are a consequence of rollers in peristaltic pumps (34, 36, 40, 41). As part of this work, we compared the endogenous function of the peristaltic pump against our lab-built pulse dampeners and a commercial pulse dampener (Fig. 2, S Figs. 1 and 2). These studies demonstrated the ability of our lab-built dampeners to generate laminar type flow in contrast to the pulsatile nature of the endogenous pump function. In addition, our dampeners showed improved performance for generation of laminar nonpulsatile flow forces compared with the commercial dampener we tested. The analysis also confirms that our custom-built dampeners are stable across at least 24 h of assay at the temperature and humidity recommended for EC culture (Fig. 2, *C*, *F*, *I*), showing that this system is suitable for use in functional assays. Therefore, modification of a peristaltic pump to include dampeners into the system allows for an affordable and adaptable method to culture cells under laminar and/or pulsatile flow conditions for the reproducible study of shear stress–mediated cellular responses *in vitro*.

Functionally, the peristaltic pump utilized has four pressure heads, allowing for side-by-side comparison of cellular behavior under pulsatile *versus* laminar flow conditions. As shown by cellular tracking experiments, cells under pulsatile flow move haphazardly and tend to end up oriented perpendicular to the direction of flow (Fig. 3, *A*–*C* and *E*; Video 5), whereas cells under laminar flow rapidly orient parallel to and move against the direction of flow (Fig. 3, *A*, *B*, *D*, *E*, Video 6). These phenotypes are consistent with those published *in vitro* and *in vivo* under developmental and nonpathogenic flow settings (5, 6, 13, 14, 15, 16, 17, 18, 21, 22, 24, 25, 26, 27, 28, 29, 31, 37, 42, 43, 44, 45, 46, 47), suggesting that this system is able to generate physiologically relevant flow forces. Finally, we confirm that ECs experiencing flow forces develop stronger junctions, as assessed by increased VE-cadherin localization at junctions compared with no flow conditions (Fig. 4). While the intensity in staining is not significantly different between cells experiencing pulsatile *versus* laminar flow, localization of VE-cadherin fingers at the junction is differentially oriented (Fig. 4, *C*–*G*), with junctional fingers aligning to be parallel to flow under laminar flow conditions (Fig. 4*F*). Similar results were described previously in EC cultures, where disturbed flow regions in a flow chamber exhibited discontinuous/more “finger”-like VE-cadherin staining, whereas in areas where the flow was laminar, the VE-cadherin staining was continuous (39, 48). Our findings reveal a similar phenomenon with actin stress fibers in response to flow, with the actin stress fibers oriented haphazardly following pulsatile flow, but oriented parallel to flow in the laminar flow conditions (Fig. 5).

Being able to reliably model physiological flow conditions allows for deeper mechanistic study of EC autonomous biology; however, it also opens up the possibility of identifying flow-regulated signals generated in ECs that alter physiology in a nonautonomous fashion. Embryonic and fetal hematopoietic stem cells (HSCs) and vascular mural cells (MCs) are two primary examples of this phenomenon. HSC development is dependent on blood flow *via* cell-intrinsic nitric oxide signaling (49). HSC development *via* the Yes-activated protein is also dependent on the mechanical forces of blood flow, as shown by Lundin *et al.* (50) using induced pluripotent stem cells grown in microfluidic culture devices. Beyond initial specification, HSCs extravasate into circulation and seed developmental niches where they complete their developmental program (reviewed by Horton *et al*. (51)). During this process, the HSCs are exposed to multiple vascular environments experiencing a variety of mechanical stresses; these stresses trigger signaling events in vascular cells that may play a role in determining ultimate hematopoietic cell fate, among other physiological processes. While there has been tremendous progress in understanding the effects of extracellular forces on HSC differentiation, our understanding of how these forces interface with the vascular niche to signal to HSCs traversing blood vessels has been limited by technical challenges.

Similarly, we have recently shown that differential sensing of blood flow forces can alter MC biology during development (52, 53). Arteries are known to acquire much greater numbers of MCs than veins across development, and elevated mechanical forces felt by the arterial vasculature is predicted to be a key driver in this process. However, how these forces are sensed by ECs and subsequently communicated to MCs is still an active area of investigation. The transcription factor Klf2 is well known to play an essential role in vascular development in response to forces generated by blood flow (44, 45, 54, 55, 56, 57, 58, 59, 60, 61, 62). In zebrafish and mice, during early vascular development, the expression of *klf2a/*Klf2 is significantly higher in veins compared with expression in the dorsal aorta, where the blood flow is pulsatory because of the dorsal aorta direct connection to the heart (43, 52, 53). We recently demonstrated that in *klf2a*-deficient zebrafish, there is a significant increase in association of MCs to the cardinal vein compared with wildtype siblings, suggesting that Klf2 might serve as a direct or indirect transcriptional repressor for MC recruitment cues (52).

These are just a few examples of anatomical adaptations that result as a response to the different types of flow forces experienced by vasculature during development or in disease. Therefore, the development of low cost and easy to use devices to model blood flow forces, such as the one described in this article, has the potential to greatly expand our understanding of the physiological effects of EC intrinsic and extrinsic signaling events.

## Experimental procedures

**Table:** 

Product	Vendor	Catalog number
Supplies required to assemble the dampeners and flow system		
Elbow Luer Connector Male	ibidi	10802
T-type connector	Nalgene	6151-0125
Traceable Silicone Pump Tubing	FisherBrand	15-078271
μ-Slide I 0.4 Luer	ibidi	80176
Plastic bonder	JBweld	50133H
10 ml Syringe Luer-Lok Tip	Becton Dickinson (BD)	302995
3 ml Syringe	BD	309656
Thin wall polypropylene tubes	Beckman	331372
30 ml HDPE bottles	Nalgene	2104-0001
Peristaltic pump and controller software	Flocel	WPX1
Supplies required to evaluate effect of dampeners		
Qdot 655 ITK organic quantum dots	Invitrogen	Q217721MP
Liquid flow sensor	Sensirion	SLF3S-1300F
Liquid flow pulsation damping kit	Sensirion	403-LFPDKIT
Reagents for immunostaining		
PFA	Sigma–Aldrich	P6148
Triton X-100	Sigma–Aldrich	T9284
BSA	Sigma–Aldrich	A7030
Goat IgG-anti-hVE-cadherin	R&D Systems	AF938
Alexa Fluro-488 donkey antigoat	Invitrogen	A11055
Hoechst 34580	Sigma–Aldrich	63493
Supplies for cell culture		
HUVEC-XL, human umbilical vein ECs	Lonza	001911027
Gelatin type B	Fisher Chemicals	G7-500
1 × M199	Gibco	11043-023
Heparin sodium salt	Sigma–Aldrich	H3393-50KU
Antibiotic–antimycotic (100×)	Gibco	15240-062
EC growth supplement	EMD Millipore	02-102
Fetal bovine serum	Gibco	16140-071

### Calculation of shear stress

Shear stress is the stress/force felt by the endothelium related to hemodynamic forces in a blood vessel. Liquid flow through a cylindrical pipe, such as blood vessels, generates a force that is parallel to the pipe wall. The shear stress applied depends on the viscosity and velocity of the flowing fluid. Normally, viscous fluids will require more force to be transported inside of a pipe and will generate more stress. In addition, higher velocities produce more force on the pipe’s walls than slow ones (63). For this platform, shear stress was calculated using the equations previously adapted by ibidi (Application Note 11. ibidi GmbH, version 6.0, February 14, 2022; https://ibidi.com/content/64-application-notes) as followed:

τ=η·κ·Φ

where τ: shear stress (dyn/cm^2^); η: dynamical viscosity (dyn⋅s/cm^2^); Φ: flow rate (ml/min); and κ: 136.6. This factor is calculated considering the viscosity of the liquid fluid—in this case, cell culture media supplemented with fetal bovine serum (∼0.0072 dyn⋅s/cm^2^) and the shear rate—which indicates the velocity of the perfused fluid in a channel (64).

### Microscopy and cell visualization

HUVEC cultures were stabilized at an initial concentration of 3000 cell/cm^2^ and maintained in standard conditions: 37 °C, 5% CO_2_, and 95% humidity until cells reached 80 to 90% confluence. Then the cells were trypsinized and reseeded on μ-Slide I 0.4 Luer slide at a final concentration of 2.5 × 10^5^ cell/cm^2^ and incubated in standard conditions for an additional 24 h before starting the flow assays.

Flow assays were carried out in an EVOS M7000 microscope, equipped with an onstage incubator to maintain standard cell culture conditions. Time-lapse images were captured in bright field at 10 × magnification (Olympus; UPlanSApo objective), every 20 min for 24 h. The videos were created by stitching the 73 frames acquired together at a rate of five frames per second. The immunostaining images were acquired at 20 × (Nikon; CFI plan Apo Lambda 20 × objective) and 60 × (Nikon; CFI plan Apo Lambda 60 × objective) with a Nikon Ti2 microscope equipped with a W1 spinning disk.

### Automated analysis of alignment parameters using ImageJ

Analysis of the effect of flow type on EC alignment was carried out using the ImageJ plugin “Directionality v2.3.0” created by Jean-Yves Tinevez (65), as follows:

Images of 2048 pixels by 1536 pixels with a resolution of 50,300 pixels/inch were transformed into 8 bit files (3 MB). The plugin was applied using the Fourier component method of analysis, with data collected from −90º to +90º and plotted in the histogram as a proportion of events with a given alignment angle per bin. Bin sizes are set at 22.5^°^, and angles of alignment are measured against the horizontal axis of the image. For this analysis, 15 images at 0 and 24 h were evaluated for both pulsatile and laminar flow.

The same method was implemented to analyze the orientation of the F-actin filaments under the same flow conditions. For this assay, eight images at 24 h after flow were evaluated for pulsatile, laminar, and no flow.

### Analysis of EC junctional markers under flow

Images of cells under different flow conditions and immunostained with VE-cadherin were evaluated using the ImageJ plugin “OrientationJ” created by Daniel Sage as follows: Images of the VE-cadherin immunostaining under no flow, laminar, and pulsatile flow were acquired with a size of 1074 microns by 629 microns with a resolution of 4.7 pixels/micron. The images were transformed to 32 bit (57 MB). VE-cadherin fingers at the upper and lower junctions of cells (Fig. S3), those parallel to the direction of the flow, were measured implementing the OrientationJ module “Quantitative Orientation Measurement” (66). The cells in each picture were selected randomly by creating a grid of squares with an area of 8337 μm^2^. A table of random numbers was then created to select the position of the cells to be measured. Using the rectangle tool in ImageJ, a Region of Interest was delimited at the points measured in each cell. The software automatically defines the orientation of the VE-cadherin fingers in the region of interest against the horizontal axis of the image. Using this method, we measured the orientation of junctional VE-cadherin fingers in 9 cells per picture, five pictures per condition.

## Data availability

All data reported in this article are available from the corresponding author upon request.

## Supporting information

This article contains supporting information.

## Conflict of interest

The authors declare that they have no conflicts of interest with the contents of this article.

## Supporting information

Video 1 **Visualization of Qdots under pulsatile flow conditions without dampeners.** Qdots were diluted 1:1000 in full culture media and flowed across the imaging slides for visualization in real-time. The video demonstrates Qdot movement under pulsatile flow conditions (the standard pump function without the pulse dampeners present) and a constant pump speed. The video was taken using the EVOS M7000 imaging system, for 21.55 s, 237 frames with a frame rate of 11 frames per second. The pump’s flow rate was reduced to 4.4 mL/min compared to the experimental condition of 15.9 mL/min to help visualization of the Qdots movement. Bar = 275 μm

Video 2 **Visualization of Qdots under laminar flow conditions with dampeners.** Qdots were diluted 1:1000 in full culture media and flowed across the imaging slides for visualization in real-time. The video demonstrates Qdot movement with dampeners located at the inlet and outlet ports of the pump head to generate laminar flow at a constant pump speed. The video was taken using the EVOS M7000 imaging system, for 21.09 s, 232 frames with a frame rate of 11 frames per second. The pump’s flow rate was reduced to 4.4 mL/min compared to the experimental condition of 15.9 mL/min to help visualization of the Qdots movement. Bar = 275 μm

Video 3 **Visualization of HUVECs under pulsatile flow conditions without dampeners.** Human umbilical vein endothelial cells (HUVECs) were cultured on a μ-Slide | 0.4 Luer slide (2.5 × 105 cell/slide) in full culture media. The video was taken using the EVOS M7000 imaging system, for 20.03 s, 621 frames with a frame rate of 31 frames per second. The pump’s flow rate was reduced to 4.4 mL/min compared to the experimental condition of 15.9 mL/min to help visualization of liquid movement. Bar = 275 μm

Video 4 **Visualization of HUVECs under laminar flow conditions with dampeners.** Human umbilical vein endothelial cells (HUVECs) were cultured on a μ-Slide | 0.4 Luer slide (2.5 × 105 cell/slide) in full culture media. The video was taken using the EVOS M7000 imaging system, for 21.10 s, 633 frames with a frame rate of 30 frames per second. The pump’s flow rate was reduced to 4.4 mL/min compared to the experimental condition of 15.9 mL/min to help visualization of liquid movement. Bar = 275 μm

Video 5 **Visualization of HUVECs under pulsatile flow conditions (without dampeners).** Human umbilical vein endothelial cells (HUVECs) were cultured on a μ-Slide | 0.4 Luer slide (2.5 × 105 cell/slide) in full culture media. The video shows a 24 h time lapse sequence, with images acquired at 20 min intervals. The cells were cultured under pulsatile flow conditions (the standard pump function without the pulse dampeners present) and a constant pump speed. Frame rate: 5 frames/sec. Bar = 275 μm

Video 6 **Visualization of HUVECs under laminar flow conditions (utilizing dampeners).** Human umbilical vein endothelial cells (HUVECs) were cultured on a μ-Slide | 0.4 Luer slide (2.5 × 105 cell/slide) in full culture media. The video shows a 24 h time lapse sequence, with images acquired at 20 min intervals. The cells were cultured under laminar flow conditions—i.e. dampeners located at the inlet and outlet ports of the pump head to generate laminar flow at a constant pump speed. Frame rate: 5 frames/sec. Bar = 275 μm

Figure S1 **Variations in the flow circuitry to evaluate the effect of the SENSIRION commercial dampener.** To evaluate the effect of the position of the SENSIRION damping tube and the addition of a restrictor to the outlet side of the flow sensor, we modified the original orientation of the system and measured flow responses. The first variation (V.1) features the addition of the restrictor to the outlet side of the flow sensor. *A*) Schematic diagram of the flow circuit variation V.1 and placement of the flow sensor before the ibidi chamber. The SENSIRION damping tube was located between the media reservoir and the flow sensor. *B*) Pulse traces collected across 15 sec of pump function, demonstrating marked pulsation of the fluid that is being flowed across the endothelial cell monolayer. *C*) Average maximum and minimum flow forces generated at 1 and 24 h of culture. The second variation (V.2) moves the sensor with the restrictor to be installed between the pump outlet and the media reservoir, the SENSIRION damping tube remained located between the media reservoir and the inlet of the ibidi chamber. *D*) Schematic diagram of the modified laminar flow circuit variation V.2. *E*) Pulse traces collected across 15 sec of pump function, demonstrating disrupted flow (18 to 0 mL/min fluctuation) across the endothelial cell monolayer. *F*) Average maximum and minimum flow forces generated at 1 and 24 h of culture. The third variation (V.3) leaves the flow sensor with the restrictor installed between the outlet of the media reservoir and the inlet of the ibidi chamber (as in A), but the SENSIRION damping tube was moved and installed between the pump outlet and the media reservoir. *G*) Schematic diagram of the modified flow circuit variation V.3. *H*) Pulse traces collected across 15 sec of pump function, demonstrating fluid pulsation across the endothelial cell monolayer. The location of the sensor and the dampener seems to have little effect on the performance of the commercial dampener system

Figure S2 **Variation of the flow system to evaluate the effect of the SENSIRION restrictor.** The addition of the restrictor to the systems showed a small reduction in the flow pulsation in the system when using the SENSIRION damping tube. We were interested in evaluating the performance of our dampeners when adding the restrictor to the outlet side of the flow sensor. *A*) Schematic diagram of the flow circuit to produce laminar flow. Our dampeners were installed at the inlet and outlet points of the peristaltic pump head. The flow sensor including the restrictor was located between the inlet of the ibidi chamber (μ-Slide I ^0.4^ Luer) and the media reservoir. *B*) Pulse traces collected across 15 sec of pump function, demonstrating minimal pulsation of the fluid that is being flowed across the endothelial cell monolayer. *C*) Average maximum and minimum flow forces generated following adaptations to the flow circuit (inclusion of homebuilt dampeners and a restrictor on the flow sensor) at 1 and 24 h of culture. The inclusion of the restrictor improved the ‘smoothness’ of laminar flow by 15% compared with our system without the restrictor

Figure S3 **Possible scenarios of VE-Cadherin junctional finger orientation in response to flow stimulus type.** Under different types of flow stimulus, cell junctions can exhibit variation in the orientation of VE-Cadherin ‘fingers’ (protrusions within the junction that interact between cells). We have observed distinctive patterns that can be described as follow: 1) when grown under conditions of no flow, endothelial cells establish dense, short, tightly packed and randomly oriented VE-cadherin fingers; 2) when exposed to pulsatory flow, endothelial cells begin to reorganize, leading to bigger intercellular spaces, and VE-cadherin junctions are longer but still randomly oriented; 3) when exposed to laminar flow, endothelial cells reorient to move/align parallel to the direction of flow, leading to tight intercellular space, and long VE-cadherin fingers that are aligned to the direction of the flow
