# Supplementary material for: Peristaltic pumps adapted for laminar flow experiments enhance in vitro modeling of vascular cell behavior
Source: J Biol Chem. 2022 Aug 19;298(10):102404. doi: 10.1016/j.jbc.2022.102404 (PMC9508572; doi:10.1016/j.jbc.2022.102404)

Abello, et al., Supplemental Figure 1

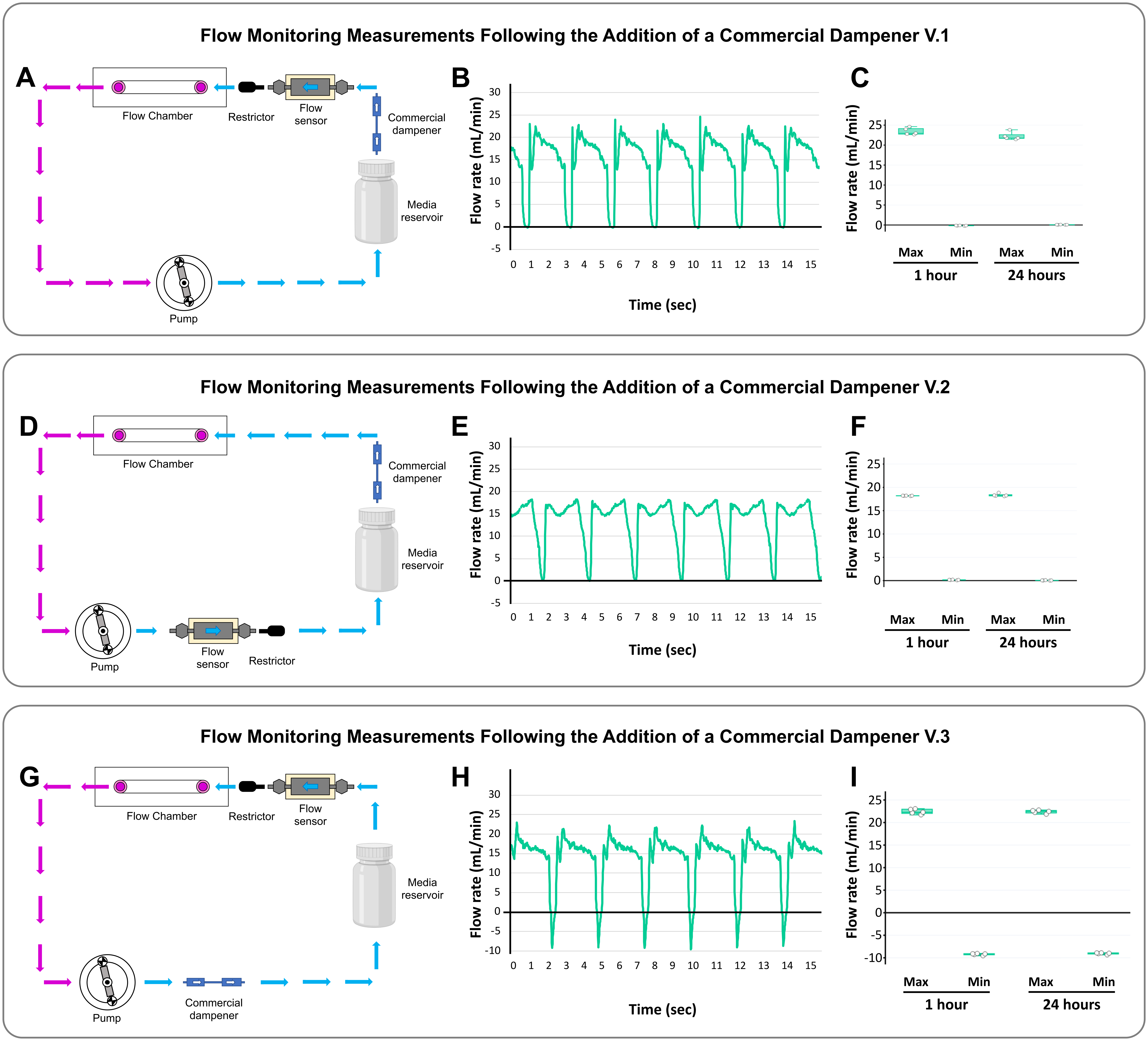

Supplement: Figure S1 — Variations in the flow circuitry to evaluate the effect of the SENSIRION commercial dampener. To evaluate the effect of the position of the SENSIRION damping tube and the addition of a restrictor to the outlet side of the flow sensor, we modified the original orientation of the system and measured flow responses. The first variation (V.1) features the addition of the restrictor to the outlet side of the flow sensor. A) Schematic diagram of the flow circuit variation V.1 and placement of the flow sensor before the ibidi chamber. The SENSIRION damping tube was located between the media reservoir and the flow sensor. B) Pulse traces collected across 15 sec of pump function, demonstrating marked pulsation of the fluid that is being flowed across the endothelial cell monolayer. C) Average maximum and minimum flow forces generated at 1 and 24 h of culture. The second variation (V.2) moves the sensor with the restrictor to be installed between the pump outlet and the media reservoir, the SENSIRION damping tube remained located between the media reservoir and the inlet of the ibidi chamber. D) Schematic diagram of the modified laminar flow circuit variation V.2. E) Pulse traces collected across 15 sec of pump function, demonstrating disrupted flow (18 to 0 mL/min fluctuation) across the endothelial cell monolayer. F) Average maximum and minimum flow forces generated at 1 and 24 h of culture. The third variation (V.3) leaves the flow sensor with the restrictor installed between the outlet of the media reservoir and the inlet of the ibidi chamber (as in A), but the SENSIRION damping tube was moved and installed between the pump outlet and the media reservoir. G) Schematic diagram of the modified flow circuit variation V.3. H) Pulse traces collected across 15 sec of pump function, demonstrating fluid pulsation across the endothelial cell monolayer. The location of the sensor and the dampener seems to have little effect on the performance of the commercial dampener system [file mmc7.pdf]
