# Supplementary figures and images for: Peristaltic pumps adapted for laminar flow experiments enhance in vitro modeling of vascular cell behavior
Source: J Biol Chem. 2022 Aug 19;298(10):102404. doi: 10.1016/j.jbc.2022.102404 (PMC9508572; doi:10.1016/j.jbc.2022.102404)

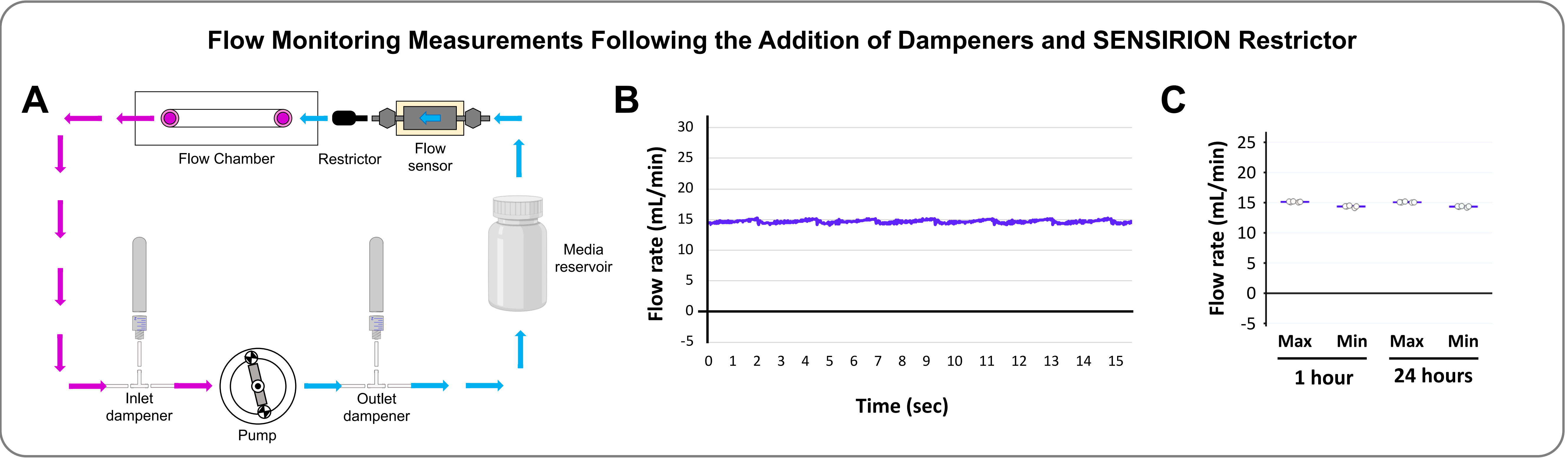

Supplement: Figure S2 — Variation of the flow system to evaluate the effect of the SENSIRION restrictor. The addition of the restrictor to the systems showed a small reduction in the flow pulsation in the system when using the SENSIRION damping tube. We were interested in evaluating the performance of our dampeners when adding the restrictor to the outlet side of the flow sensor. A) Schematic diagram of the flow circuit to produce laminar flow. Our dampeners were installed at the inlet and outlet points of the peristaltic pump head. The flow sensor including the restrictor was located between the inlet of the ibidi chamber (μ-Slide I 0.4 Luer) and the media reservoir. B) Pulse traces collected across 15 sec of pump function, demonstrating minimal pulsation of the fluid that is being flowed across the endothelial cell monolayer. C) Average maximum and minimum flow forces generated following adaptations to the flow circuit (inclusion of homebuilt dampeners and a restrictor on the flow sensor) at 1 and 24 h of culture. The inclusion of the restrictor improved the ‘smoothness’ of laminar flow by 15% compared with our system without the restrictor [file mmc8.pdf]
