# Supplementary material for: Peristaltic pumps adapted for laminar flow experiments enhance in vitro modeling of vascular cell behavior
Source: J Biol Chem. 2022 Aug 19;298(10):102404. doi: 10.1016/j.jbc.2022.102404 (PMC9508572; doi:10.1016/j.jbc.2022.102404)

Abello, et al., Supplementary Figure 3

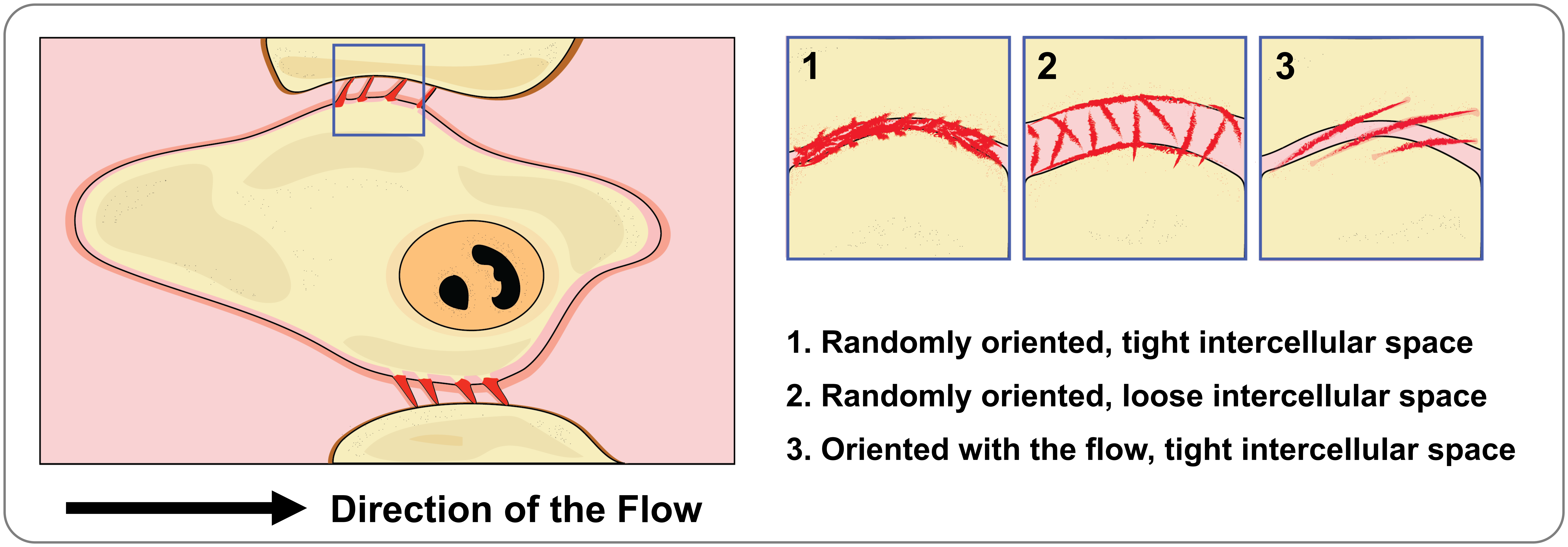

Supplement: Figure S3 — Possible scenarios of VE-Cadherin junctional finger orientation in response to flow stimulus type. Under different types of flow stimulus, cell junctions can exhibit variation in the orientation of VE-Cadherin ‘fingers’ (protrusions within the junction that interact between cells). We have observed distinctive patterns that can be described as follow: 1) when grown under conditions of no flow, endothelial cells establish dense, short, tightly packed and randomly oriented VE-cadherin fingers; 2) when exposed to pulsatory flow, endothelial cells begin to reorganize, leading to bigger intercellular spaces, and VE-cadherin junctions are longer but still randomly oriented; 3) when exposed to laminar flow, endothelial cells reorient to move/align parallel to the direction of flow, leading to tight intercellular space, and long VE-cadherin fingers that are aligned to the direction of the flow [file mmc9.pdf]
